# Socio-cultural determinants of physical activity across the life course: a ‘Determinants of Diet and Physical Activity’ (DEDIPAC) umbrella systematic literature review

**DOI:** 10.1186/s12966-017-0627-3

**Published:** 2017-12-20

**Authors:** Lina Jaeschke, Astrid Steinbrecher, Agnes Luzak, Anna Puggina, Katina Aleksovska, Christoph Buck, Con Burns, Greet Cardon, Angela Carlin, Simon Chantal, Donatella Ciarapica, Giancarlo Condello, Tara Coppinger, Cristina Cortis, Marieke De Craemer, Sara D’Haese, Andrea Di Blasio, Sylvia Hansen, Licia Iacoviello, Johann Issartel, Pascal Izzicupo, Martina Kanning, Aileen Kennedy, Fiona Chun Man Ling, Giorgio Napolitano, Julie-Anne Nazare, Camille Perchoux, Angela Polito, Walter Ricciardi, Alessandra Sannella, Wolfgang Schlicht, Rhoda Sohun, Ciaran MacDonncha, Stefania Boccia, Laura Capranica, Holger Schulz, Tobias Pischon, on behalf of the DEDIPAC consortium

**Affiliations:** 1 Molecular Epidemiology Group, Max Delbrück Center for Molecular Medicine in the Helmholtz Association (MDC), Berlin-Buch, Robert-Roessle-Strasse 10, 13125 Berlin, Germany; 2 Institute of Epidemiology I, Helmholtz Zentrum München - German Research Center for Environmental Health, Neuherberg, Germany; 3 Section of Hygiene - Institute of Public Health; Università Cattolica del Sacro Cuore, L.go F. Vito, 1 –, 00168 Rome, Italy; 4 Leibniz Institute for Prevention Research and Epidemiology – BIPS, Bremen, Germany; 5 Department of Sport, Leisure and Childhood Studies, Cork Institute of Technology, Cork, Munster, Ireland; 6 Department of Movement and Sports Sciences, Ghent University, Ghent, Belgium; 7 Department of Physical Education and Sport Sciences, University of Limerick, Limerick, Ireland; 8 Centre de Recherche en Nutrition Humaine Rhône-Alpes, CarMeN INSERM U1060, University of Lyon1, Lyon, France; 9 Council for Agricultural Research and Economics - Research Centre for Food and Nutrition, Rome, Italy; 10 Department of Movement, Human and Health Sciences, University of Rome Foro Italico, Rome, Italy; 11 Department of Human Sciences, Society, and Health, University of Cassino and Lazio Meridionale, Cassino, Italy; 12 Department of Medicine and Aging Sciences, ‘G. d’Annunzio’ University of Chieti-Pescara, Chieti-Pescara, Italy; 13 Department for Sport and Exercise Sciences, University of Stuttgart, Stuttgart, Germany; 14 Department of Epidemiology and Prevention, IRCCS Istituto Neurologico Mediterraneo: NEUROMED, Pozzilli, Italy; 15 School of Health and Human Performance, Multisensory Motor Learning Lab., Dublin City University, Dublin, Ireland; 16 Department for Sport Sciences, University of Konstanz, Konstanz, Germany; 17 Centre for Preventive Medicine, School of Health and Human Performance, Dublin City University, Dublin, Ireland; 18 Institute of Sport, Exercise & Active Living, Victoria University, Melbourne, Australia; 19 Luxembourg Institute of Socio-Economic Research (LISER), Esch/Alzette, Luxembourg; 20 Section of Hygiene - Institute of Public Health; Università Cattolica del Sacro Cuore, Fondazione Policlinico Universitario “Agostino Gemelli”, L.go F. Vito, 1 –, 00168 Rome, Italy; 21 Italian National Institute of Health (Istituto Superiore di Sanita - ISS), Rome, Italy; 22 Icahn School of Medicine at Mount Sinai, 17 East 102 St, New York, USA; 23 Charité Universitätsmedizin Berlin, Berlin, Germany; 24 DZHK (German Center for Cardiovascular Research), partner site Berlin, Berlin, Germany

**Keywords:** Socio-cultural determinants, Physical activity, Life course, Children, Adolescents, Adults, Umbrella systematic literature review

## Abstract

**Objective:**

Regular physical activity (PA) reduces the risk of disease and premature death. Knowing factors associated with PA might help reducing the disease and economic burden caused by low activity. Studies suggest that socio-cultural factors may affect PA, but systematic overviews of findings across the life course are scarce. This umbrella systematic literature review (SLR) summarizes and evaluates available evidence on socio-cultural determinants of PA in children, adolescents, and adults.

**Methods:**

This manuscript was drafted following the recommendations of the ‘Preferred Reporting Items for Systematic reviews and Meta-Analyses’ (PRISMA) checklist. The MEDLINE, Web of Science, Scopus, and SPORTDiscus databases were searched for SLRs and meta-analyses (MAs) on observational studies published in English that assessed PA determinants between January 2004 and April 2016. The methodological quality was assessed and relevant information on socio-cultural determinants and any associations with PA was extracted. The available evidence was evaluated based on the importance of potential determinants and the strength of the evidence.

**Results:**

Twenty SLRs and three MAs encompassing 657 eligible primary studies investigated potential socio-cultural PA determinants, with predominantly moderate methodological quality. Twenty-nine potential PA determinants were identified that were primarily assessed in children and adolescents and investigated the micro-environmental home/household level. We found probable evidence that receiving *encouragement from significant others* and *having a companion for PA* were associated with higher PA in children and adolescents, and that parental *marital status (living with partner)* and experiencing *parental modeling* were not associated with PA in children. Evidence for the other potential determinants was limited, suggestive, or non-conclusive. In adults, quantitative and conclusive data were scarce.

**Conclusions:**

A substantial number of SLRs and MAs investigating potential socio-cultural determinants of PA were identified. Our data suggest that receiving social support from significant others may increase PA levels in children and adolescents, whereas parental marital status is not a determinant in children. Evidence for other potential determinants was limited. This was mainly due to inconsistencies in results on potential socio-cultural determinants of PA across reviews and studies.

**Trial registrations:**

This umbrella SLR was recorded on PROSPERO (Record ID: *CRD42015010616*).

**Electronic supplementary material:**

The online version of this article (10.1186/s12966-017-0627-3) contains supplementary material, which is available to authorized users.

## Background

Lack of physical activity (PA) is an established risk factor for numerous chronic diseases and premature death, whereas regular PA reduces disease and mortality risk [1–3]. For adults (i.e., 18–64 years) and older adults (i.e., ≥65 years), the World Health Organization (WHO) recommends at least 150 min of moderate or 75 min of vigorous PA per week to prevent non-communicable diseases, while children and adolescent between 5 and 17 years should accumulate at least 60 min of moderate-to-vigorous activity [2]. Nevertheless, 23% of adults globally, up to one third of European adults, and a vast majority of children and adolescents in Europe and worldwide are not sufficiently active to meet these recommendations [4–6]. Low PA accounts for a huge, but avoidable disease burden and is among the five leading risks for mortality in the world, responsible for 5.5% of deaths globally [3, 7]. In addition, it is among the seven leading risk factors for disability-adjusted life years (DALYs), responsible for 3.5% of DALYs in the WHO European Region [8].

Research into determinants (causally related factors) and correlates (associated factors) of PA have increased in the last decade, and several factors have been identified to be purportedly related to PA, including socio-cultural factors [9–12]. Socio-cultural determinants of PA are defined as ‘community's or society's attitudes, beliefs, and values related to health behaviour’ that might have a ‘powerful effect on the behaviour of individual members of the community group’ [13]. However, systematic overviews on socio-cultural determinants of PA are scarce and mainly focus on specific age ranges, neglecting the possibility to evaluate the impact of socio-cultural PA determinants in different age groups [9, 10].

The aim of this umbrella systematic literature review (SLR) was to provide an overview, compilation, and evaluation of the available evidence from published SLRs and meta-analyses (MAs) of primary observational studies assessing socio-cultural determinants of PA in children, adolescents, and adults.

## Materials and methods

The European Commission has initiated the ‘Joint Programming Initiative A Healthy Diet for a Healthy Life’ aiming to enhance cooperation, to pool knowledge, and to engage in a common research agenda to finally promote healthy lifestyles across Europe [14]. As first act, the ‘**DE**terminants of **DI**et and **P**hysical **AC**tivity (DEDIPAC) **K**nowledge **H**ub (KH)’ was launched in 2013 as a multi-disciplinary collaboration of experts, organizations, and consortia across 12 European countries [15]. One of the aims of the DEDIPAC KH was to assess determinants of PA across the life course. The DEDIPAC KH coordinated seven umbrella SLRs (i.e., reviews that assemble together several systematic reviews on the same condition [16]) on the evidence on biological, psychological, behavioural, physical, socio-cultural, economic, and policy determinants of PA [15]. Diet was addressed separately [17].

The seven manuscripts were drafted following recommendations of the ‘**P**referred **R**eporting **I**tems for **S**ystematic reviews and **M**eta-**A**nalyses’ (PRISMA) checklist [18]. The protocol applied to all seven DEDIPAC umbrella SLRs was recorded on PROSPERO (Record ID: *CRD42015010616*), the international prospective register of systematic reviews [19].

### Search strategy and eligibility criteria

To identify eligible SLRs and MAs investigating determinants of PA in different age groups, a systematic online search limited to English publications was conducted in MEDLINE, Web of Science, Scopus, and SPORTDiscus databases. To avoid duplications of the earliest individual studies included in the SLRs and MAs, the search was limited to publications between January, 1st, 2004 and April, 30th, 2016. The decision on the cut-off date was made since the seven umbrella SLRs were initiated in 2014 and the DEDIPAC KH aimed to encompass a 10-years publication period [15]. In 2016, prior to finalizing the seven umbrella SLRs, the literature search was updated to also include publications in 2015 and 2016, and, thus, to encompass the lifetime of the DEDIPAC project. For all seven umbrella SLRs, the same search strategy (Additional file 1) and eligibility criteria were used. SLRs or MAs of observational primary studies on the association between any variable and PA, exercise, or sport as main outcome were initially included. Sedentary behaviour was not included in the current umbrella SLR as it was addressed separately [20, 21]. The following exclusion criteria were applied: i) SLRs and MAs of intervention studies; ii) SLRs and MAs that focused on specific disease groups; iii) umbrella SLRs.

### Selection process

The identified articles were arranged alphabetically and distributed among the 15 partners of the DEDIPAC KH. For each partner, two reviewers independently screened the titles, abstracts, and full texts of assigned articles and assessed them for eligibility. Before final inclusion or exclusion, a common decision had to be reached; any uncertainty and disagreement was resolved by consulting three further authors to reach consensus (SB, LC, AP).

The SLRs and MAs judged eligible were referred to as ‘reviews’. PA was classified broadly to include the whole spectrum, from unstructured daily activities to exercise and competitive sports, independently from frequency, duration, or intensity.

### Quality assessment of SLRs

Methodological quality of eligible SLRs was evaluated using a slightly modified version of ‘**A M**ea**S**urement **T**ool to **A**ssess systematic **R**eviews’ (AMSTAR) [22, 23]. AMSTAR requires as one criterion a conflict of interest statement in the published SLR, as well as in the studies included in the SLR; for this umbrella SLR it was sufficient if this statement was provided in the published SLR.

Eligible SLRs were distributed among the DEDIPAC KH partners and quality was independently assessed by two reviewers from each partner; any uncertainty and disagreement was resolved by consensus by three further authors (SB, LC, AP). AMSTAR criteria were scored 1 if they were fulfilled by the SLR or 0 if not applicable, not fulfilled, or could not be answered based on the information provided by the SLR. The summed quality score was classified as weak (sum quality score ≤ 3), moderate (4 to 7), or strong (≥8).

### Data extraction

The following data were independently extracted by two reviewers from each partner: author and year of publication, type of review (SLR or MA); total number of primary studies (all studies included within the review) and number of primary studies that focused on socio-cultural determinants (in the following defined as ‘eligible primary studies’). Subsequently, for each eligible primary study, information on the study (e.g., study design, age), PA outcome (e. g., overall or moderate PA), and year of publication was extracted. Study design of eligible primary studies was classified as ‘quantitative cross-sectional’, ‘quantitative longitudinal’ (including follow-up information), ‘qualitative’, or ‘other’. Only quantitative eligible primary studies were systematically analysed. Further, information on the socio-cultural determinant(s) assessed in the eligible primary studies was extracted. Additionally, the overlap of eligible primary studies between reviews was identified. Some reviews provided results for eligible primary studies, others for sub-samples of eligible primary studies, for example, separately for sexes or PA outcomes; collectively, these are defined as ‘eligible samples’ (either eligible primary studies or eligible sub-samples) and form the basis for this umbrella SLR. The number of positive, negative, null, or indecisive associations reported for eligible samples with regard to specific determinants was extracted.

Since eligible primary studies included in the reviews were of cross-sectional as well as longitudinal design, in the following, the term ‘potential determinant’ is used to encompass correlates (associated factors identifiable via cross-sectional studies) and determinants (causally related factors, requiring longitudinal analyses) of PA.

### Categorization of included socio-cultural determinants of PA and age groups

Following the ‘**AN**alysis **G**rid for **E**nvironments **L**inked to **O**besity’ (ANGELO) framework, identified potential socio-cultural determinants were grouped into the ‘home/household’, ‘educational institutions’, ‘workplace’, or ‘neighbourhood’ level, representing the micro-environment of individuals’ interaction, or the ‘city/municipality/region/country’ level, representing the macro-environment [13].

Similarly or equally defined potential determinants reported in the reviews were combined; for example, ‘parental support’ and ‘encouragement from parents’ were combined to ‘*encouragement from significant others*’. Where suitable, individual potential determinants were grouped into broader categories to facilitate the structuring (e.g., *encouragement from significant others*, *having a companion for PA*, *parental modeling*, and *parental watching* were assigned to *supportive behaviour from significant others*, but were individually evaluated). If necessary, the direction of a reported association between a potential determinant and PA in the published reviews was inverted to meet the defined direction of association of potential determinants.

Findings were assigned to ‘children’, if the reported mean age or age range of eligible primary studies was <12 years, to ‘adolescents’ if 12 to ≤18 years, to ‘children and adolescents’ for populations aged ≤18 years, and to ‘adults’ for ages >18 years.

### Evaluation of the importance of determinants and strength of the evidence

Data extracted for potential determinants were summarized and evaluated by applying two slightly modified grading scales [24]. The first grading scale evaluated the ‘*importance of a potential determinant’* and refers to the number of eligible samples showing a positive, negative, or null association [24]. For MAs, significant associations or non-significant associations with effect sizes >0.3 are defined as a positive or negative association, depending on the reported direction [24]; otherwise, the finding was counted as null association. The importance of a potential determinant was scored between ‘++’ (highest level of importance for a positive or negative association) to ‘--’ (highest level of importance for no association, Table 1).

**Table 1:** Importance of a potential determinant and strength of the evidence [24, 25]

Importance of a potential determinant^a^			
	association across primary samples		
	%	direction	
++	100	positive or negative	
+	>75	positive or negative	
0	≤75	positive or negative and	
	≤75	no association	
–	>75	no association	
––	100	no association	
Strength of the evidence^b^			
	‘sufficient evidence’		‘consistency’
	reviews	independent cohorts	across primary samples
	n	n	%
Ce	≥3	≥2	100
Pe	≥2	≥2	>75
Ls	≥1	≥1	>66
Lnc	≥1	0	≤66

*Ce* Convincing evidence, *Pe* Probable evidence, *Ls* Limited, suggestive evidence, *Lnc* Limited, non-conclusive evidence

^a^Importance was evaluated based on the proportion of study that reported a positive or negative association between a potential determinant and PA. E.g., a potential determinant was scored ‘++’ if 100% of eligible samples reported either a positive or a negative association with PA

^b^Strength of the evidence was evaluated based on the number of reviews, the reported study design of eligible primary studies, and the consistency across primary samples. For each level of evidence, each criterion for number of reviews, study design, and consistency had to be fulfilled. E.g., there was ‘convincing evidence’ (Ce, highest level of evidence), if the results were: (1) based on a substantial number of reviews (here defined as ≥3 SLRs, [70]) including data of different study designs and (2) based on at least two independent primary cohort studies and, (3) showed a consistent association with PA (here defined as 100% of eligible samples reported associations to be in the same direction)

The second grading scale was based on modified recommendations of the World Cancer Research Fund [24, 25]. It evaluated the ‘*strength of the evidence*’ based on the number of reviews, the reported study design of eligible primary studies, and the consistency across primary samples (Table 1) [24, 25].

Qualitative results of reviews were not included in the grading of the importance of potential determinants or strength of the evidence, but were reported narratively to complete and supplement the results found for quantitative primary studies where suitable.

## Results

### SLRs and MAs selection process

**Fig. 1:**
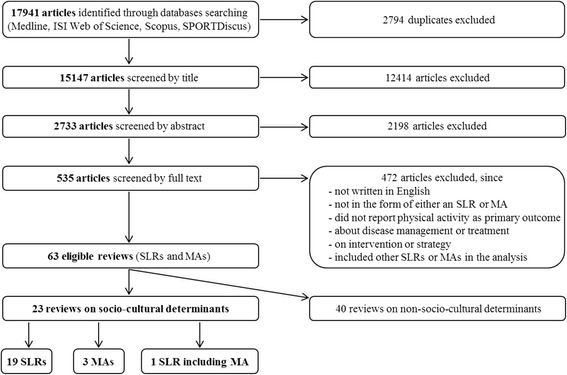
Flowchart of the online literature research by database. Results of the online literature search on systematic literature reviews (SLRs) and meta-analyses (MAs) of observational primary studies investigating potential determinants of physical activity published in English between January, 1st, 2004 and April, 30th, 2016 and the final selection of eligible reviews

In total, 17,941 articles were initially identified during the systematic literature search (Fig. 1). After elimination of duplicates, and screening of titles, abstracts, and full texts, 23 reviews were eligible for the present umbrella SLR [26–48], including 19 SLRs [26–37, 39, 41, 42, 44–47], three MAs [40, 43, 48], and one combined SLR/MA [38] (Fig. 1).

### Quality assessment of the included SLRs

The quality assessment was performed for the 20 included SLRs (Additional file 2). Of these, 14 were evaluated as being of moderate [26, 28, 29, 31–35, 38, 39, 42, 44, 45, 47] and six as being of weak quality [27, 30, 36, 37, 41, 46].

### Characteristics of the included reviews and eligible primary studies

The characteristics of the 23 included reviews comprising a total of 657 eligible primary studies are summarized in Table 2. Two reviews focused exclusively on potential socio-cultural determinants [37, 43], whereas the others also assessed other potential PA determinants. In most reviews, the eligible primary studies came from multiple continents. The majority was conducted in North-America (64.1%) and Europe (21.8%), while few were included from Asia (2.6%) and South America (0.7%). The study design was provided for 461 (70.2%) of the 657 eligible primary studies [28, 29, 31–39, 43–48]; of these 461 eligible primary studies, the greatest portion (75.9%) were classified as quantitative cross-sectional [28, 31, 33–36, 38, 39, 43, 44, 46–48] followed by quantitative longitudinal (23.2%), with follow-up periods, if reported, between 8 weeks to 13 years [29, 31–38, 43, 45–48]. The sample size of eligible primary studies ranged from 8 [26] to 80,944 [32] and the total sample size per review ranged from 350 [28] to 228,587 [32].

**Table 2:** Characteristics of the eligible primary studies

	Eligible primary studies						
Author, Date (type of review) [Ref]	n	Continent/s (n)	Study design (n)	Total sample size (sample range)	Age range or mean (years)	Gender (female, % range)	Physical activity (PA) outcome
Babakus WS, 2012 (SLR) [28]	12	Europe (9), North America (2), Australia/Oceania (1)	N. A. qualitative	587 (8–127)^a^	16–70+	N.A. to 100	overall PA
Beets MW, 2010 (SLR) [29]	39	N. A.	N. A. quantitative and qualitative	N. A.	<18^b^	N. A.	overall PA
Coble JD, 2006 (SLR) [30]	1	North America (1)	cross-sectional (1)	350	20–50	100	overall PA
Craggs C, 2011 (SLR) [31]	19	North America (14), Europe (3), Australia/Oceania (1), Asia (1)	longitudinal (19; follow-up: 4 months - 7 years)	37,518 (28–12,812)	children: 4–9 and 10-13^b^, adolescents: 13.1–16.0	N.A. to 100	change in overall PA
De Craemer M, 2012 (SLR) [32]	12	N. A.	N. A.	N. A.	4–6 or results on preschoolers^b^	N. A.	total PA, MVPA, active transportation
Edwardson CL, 2010 (SLR) [33]	86^c^	North America (54), Europe (23), Australia/Oceania (7), Asia (2)	cross-sectional (75), longitudinal (11; follow-up: 20 months - 12 years)	N. A.	children: 6-11^b^, adolescents: 12-18^b^	0–100	MVPA, overall PA, leisure-time PA, organized PA, steps per day, PA frequency and intensities
Engberg E, 2012 (SLR) [34]	11	North America (8), Australia/Oceania (3)	longitudinal (11; follow-up: 2–10 years)	228,587 (558–80,944)	≥18	0–100	change in overall PA, in fitness, and in participation in exercise
Ferreira I, 2006 (SLR) [35]	96^c^	North America (75), Europe (18), Australia/Oceania (3)	cross-sectional (86), longitudinal (10; follow-up: 8 weeks - 3 years)	N. A. (<100 to ≥5000)	children: 3-12^b^, adolescents: 13-18^b^	N.A. to 100	overall PA
Gustafson SL, 2006 (SLR) [36]	31	North America (26), Europe (5)	cross-sectional (26), longitudinal (5; follow-up: N. A.)	25,908 (30–7320)	age range: 4–18, mean age: 10.9^a^	N.A. to 100	overall PA
Hinkley T, 2008 (SLR) [37]	7	North America (7)	cross-sectional (6), longitudinal (1; follow-up: N. A.)	1095 (30–347)	age range: 3–7, mean age: 3.8	N. A.	overall PA
Maitland C, 2013 (SLR) [38]	8	Asia (2), Australia/Oceania (2), Europe (2), North America (2)	cross-sectional (6), longitudinal (2; follow-up: N. A.)	8105 (62–2660)	11.6	N. A.	overall PA, MVPA
Maturo CC, 2013 (SLR) [39]	81	North America (43), Europe (21), Australia/Oceania (9), Asia (5), multiple, continents (2), South America (1)	N. A. quantitative (67), longitudinal (14; follow-up: N. A.)	123,888 (20–31,202)*	age range: 8–21, Grade 1–12	0–100	overall PA, PA intensity (not further specified)
Mitchell J, 2012 (SLR, MA) [40]	12	North America (8), Australia/Oceania (3), South America (1)	cross-sectional (8), longitudinal (4; follow-up: 1–9 years)	1692 (30–331)	2-7^b^	N. A.	overall PA
Olsen JM, 2013 (SLR) [41]	13	North America (13)	cross-sectional (2), quantitative, descriptive (2), N. A. quantitative (3), N. A. qualitative (4), N. A. quantitative and qualitative (1), multiple, descriptive, explanatory case study (1)	6951 (17–2338)	20–65+^a^	100	overall PA
Pugliese J, 2007 (MA) [42]	29	N. A.	N. A. quantitative	21,632 (21–8834)	2.5–15.5	N. A.	overall PA
Ridgers ND, 2012 (SLR) [43]	7	Australia/Oceania (5), Europe (1), North America (1)	N. A.	N. A.	children: 5-12^b^, adolescents: 13-18^b^	N. A.	overall PA during school recess
Siddiqi Z, 2011 (SLR) [44]	22	N.A. (African Americans were included)	N. A. qualitative	797 (14–71)	≥18	48-100^a^	overall PA
Singhammer J, 2015 (MA) [45]	9	North America (4), Europe (3), Australia/Oceania (2)	cross-sectional (7), longitudinal (2; follow-up: N. A.)	11,159 (200–2458)	6–18	N. A.	overall PA
Stanley RM, 2012 (SLR) [46]	8	North America (5), Australia/Oceania (2), Europe (1)	cross-sectional (7), questionnaire validation study (1)	N. A.	10.7	N. A.	school break time overall PA, after-school overall PA
Uijtdewillingen L, 2014 (SLR) [47]	6^c^	North America (3), Europe (2), Australia/Oceania (1)	longitudinal (6; follow-up: 1–13 years)	21,163 (152–12,812)	children: 5.5, adolescents: 8.5	40–100	overall PA
Van der Horst K, 2007 (SLR) [48]	19^c^	N. A.	cross-sectional (16), longitudinal (2; follow-up: N. A.)	N. A.	children: 4-12^b^, adolescents: 13-18^b^	N. A.	overall PA
Wendel-Vos W, 2007 (SLR) [49]	24	North America (16), Australia/Oceania (6), Asia (1), Europe (1)	cross-sectional (22), longitudinal (2; follow-up: N. A.)	74672^a^ (146–29,135)	≥18	N.A. to 100	general PA, moderate PA, vigorous PA/ sports, MVPA, commuting activities, walking, neighbourhood walking
Yao CA, 2015 (MA) [50]	106	North America (62), Europe (28), Australia/Oceania (11), Asia (3), South America (2)	cross-sectional (88), longitudinal (18; follow-up: 8 months - 9 years)	163,215 (14–68,288)	2.5-18^b^	N.A. to 100	overall PA

*MA* Meta-Analysis, *MVPA* Moderate-to-Vigorous Physical Activity, *N. A.* Not Available, *PA* Physical Activity, *SLR* Systematic Literature Review

^a^No information for one study

^b^Information from inclusion criteria

^c^Some primary studies included in both children and adolescents

**Fig. 2:**
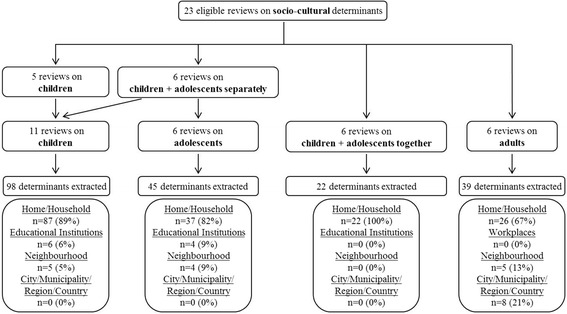
Flowchart of determinant extraction and categorization. Results of the extraction of potential socio-cultural determinants of physical activity based on the 23 included reviews for the different age groups. Potential determinants were assigned to micro- and macro-environmental levels based on the ANGELO framework [13]

Five reviews reported data on children only [30, 35, 36, 38, 44], six separately on children and adolescents [29, 31, 33, 41, 45, 46], and another six (including all MAs) on children and adolescents together [27, 34, 37, 40, 43, 48] (Fig. 2). Six reviews reported on adults [26, 28, 32, 39, 42, 47]. Of these, four examined subgroups of the general population; i.e. South Asian women with an immigrant background [26], Native Americans [28], rural women [39], and African American adults [42]. Across the 23 included reviews, 574 eligible primary studies on children and/or adolescents and 83 on adults, respectively, were originally identified. In children and/or adolescents, 23.0% of eligible primary studies were included multiple times in two to seven reviews; 4.8% of eligible primary studies on adults were included in two reviews.

### PA outcomes

Most reviews assessed a variable representing overall PA as outcome to examine determinants of PA, comprising general PA measures investigated in the eligible primary studies, like ‘total PA’, ‘overall PA’, or ‘exercise’ [26–28, 31, 33–40, 42, 43, 45, 46, 48] (Table 2). In contrast, few reviews focused on specific PA outcomes, with four reviews analysing moderate-to-vigorous PA [30, 31, 36, 47], two reviews examining moderate PA [31, 47], and two others examining change in overall PA [29, 32]. Further PA outcomes (e.g., leisure-time PA) were assessed in individual reviews [30–32, 37, 41, 44, 47]. As described, results on all PA outcomes originally investigated in the eligible reviews were combined to ‘PA’ in the present umbrella SLR to comprehensively summarize the evidence.

### Categorization of included socio-cultural determinants of PA

Initially, 98 mutually not exclusive potential socio-cultural determinants were extracted in children, 45 in adolescents, 22 in children and adolescents studied together, and 39 in adults (Fig. 2). After harmonization of terminology, 29 potential socio-cultural determinants were retained across all ages. These were assigned to the micro-environmental house/household (18 potential determinants), educational institutions (five potential determinants), and neighbourhood level (four potential determinants), or to the macro-environmental city/municipality/region/country level (two potential determinants) (Additional file 3). The home/household level included: *family composition*, *significant others’ health status, supportive behaviour from significant others, social norms, significant others’ PA*, *participation in organized sports,* and *involvement of social contact*. The educational institutions level included: *supportive behaviour at school, teacher specific educational level,* and *PA level at school (teacher PA)*. The neighbourhood level included: *seeing people exercise*, *society composition (young society)*, *social inclusion and acculturation*, and *neighbourhood satisfaction*. At the city/municipality/region/country level, *cultural climate* and *religion* were assessed (Additional file 3).

While the majority of identified socio-cultural determinants belonged to the home/household level, potential determinants of the city/municipality/region/country level were only investigated in qualitative eligible primary studies in adults (Fig. 2, Table 3).

**Table 3:** Summary of the results on the importance of potential determinants and the strength of the evidence

	**Children** (age range or mean age < 12 years)	**Adolescents** (age range or mean age ≥ 12 to ≤18 years)	**Children and adolescents** (age range or mean age ≤ 18 years)	**Adults** (age range or mean age > 18 years)
**potential determinant**	**evidence for an association with PA**			
**MICRO-ENVIRONMENT**				
A Home/Household				
1. Family composition				
** - Marital status (living with partner, yes vs. no)**	-. Pe [31, 32, 35, 48]	-, Ls [35]	0, Ls [36, 45]	0, Lnc [33] [41]
- Having siblings (number or yes vs. no)	0, Lnc [31, 32]			
- Having dogs (yes vs. no)	-, Lnc [32, 35]			
- Number of children in household (high vs. low; yes vs. no)	- -, Lnc [35]	- -, Lnc [35]		[41, 44]
- Family demands (yes vs. no)				[28, 41, 44]
2. Significant others’ health status (impaired vs. not impaired)				
- Parental BMI/waist circumference	-, Ls [31, 32]			
- Maternal depression	++, Ls^a^ [40]			
3. Supportive behaviour from significant others (yes vs. no)				
** - Encouragement from significant others**	0, Ls [31–33, 35, 37, 38, 40, 46, 48]	0, Lnc [31, 33, 35, 43, 48]	+, Pe^b^ [29, 36, 39, 42, 50]	0, Lnc [49] [28, 41, 44]
** - Having a companion for PA (yes vs. no)**	0, Lnc [31–33]	-, Ls [33, 47]	+, Pe^b^ [29, 39, 50]	0, Lnc [49]
** - Parental modeling**	-, Pe [31, 32]		++, Lnc^b^ [42]	
- Parental watching (presence, observation)	-, Lnc [33, 46]		0, Lnc [29, 50]	
4. Social norms (yes vs. no)				
- Awareness of PA	0, Ls [31–33, 35, 40]	0, Lnc [31, 33, 35]	- -, Lnc [29, 50]	
- Physician advices				[44]
- Parental concern about the environment	++, Lnc^a^ [40]			
- Familial interaction and social influences	0, Lnc [32, 37, 46]	0, Ls [48]		
5. Significant others’ PA (high vs. low)	0, Lnc [31–33, 35, 37, 38, 40, 46–48]	0, Lnc [31, 33, 35, 47, 48]	0, Ls [36, 39, 50]	[44]
6. Participation in organized sports (yes vs. no)	0, Ls [31, 32, 35, 37, 46]			[44]
7. Involvement of social contact (yes vs. no)	++, Lnc^b^ [32, 37]	- -, Ls [31]		0, Lnc [49] [41]
B Educational Institutions				
1. Supportive behaviour at school (yes vs. no)				
- Encouragement at school	- -, Lnc [35]	0, Ls [35]		
- Teacher management (organization of activities)	- -, Lnc [43]			
- Teacher watching (presence, observation)	0, Lnc [43, 46]			
2. Teacher specific educational level (yes vs. no)	++, Lnc^b^ [35]			
3. PA level at school (teacher PA, high vs. low)	- -, Lnc [35]	-, Lnc [35]		
C Neighbourhood				
1. Seeing people exercise (yes vs. no)		- -, Lnc [35]		0, Lnc [30, 49] [41]
2. Society composition (young society, yes vs. no)	++, Lnc^b^ [32]	- -, Lnc [35]		
3. Social inclusion and acculturation (yes vs. no)	0, Lnc [32, 35]	0, Ls [35]		
4. Neighbourhood satisfaction (yes vs. no)	++, Lnc^b^ [35]	- -, Lnc [35]		-, Lnc [49]
**MACRO-ENVIRONMENT**				
D City/Municipality/Region/Country				
1. Cultural climate				[28, 44]
2. Religion				[28, 41]

*BMI* Body Mass Index, *PA* Physical Activity, *Ce* Convincing evidence, *Pe* Probable evidence, *Ls* Limited, suggestive evidence, *Lnc* Limited, non-conclusive evidence

^a^Negative association

^b^Positive association

Italics: qualitative studies eligible results are not included in this grading of the evidence

Bold: determinants with probable evidence discussed in manuscript

### Importance of socio-cultural determinants of PA and strength of the evidence

None of the associations of potential socio-cultural determinants and PA assessed was evaluated as possessing convincing evidence (Table 3, Additional file 4).

Among the potential *family composition* determinants*,* there was probable evidence for no association between parental *marital status (living with partner)* and PA in children (−, Pe [29, 30, 33, 46]); 86% of all eligible primary samples reported a null result (Table 3, Additional file 4). In adolescents based on one review only, limited, suggestive evidence was found that parental *marital status (living with partner)* is not associated with PA (−, Ls [33]); 79% of all eligible samples showed no association. Where children and adolescents were studied together, there was limited, suggestive evidence for no association between parental *marital status (living with partner)* and PA (0, Ls [34, 43]); 67% of all eligible samples showed null results. For adults, there was limited, non-conclusive evidence (0, Lnc [32]) though 64% of eligible samples showed a negative association between *marital status (living with partner)* and PA (0, Lnc [32]); one qualitative eligible primary study reported being married as associated with PA in adults, but the direction of the association was not specified [39].

Further, there was probable evidence for three determinants belonging to the *supportive behaviour from significant others* determinants (Table 3, Additional file 4). Firstly, receiving *encouragement from significant others* was positively associated with PA in children and adolescents (+, Pe [27, 34, 37, 40, 48]). When analysed separately, evidence was limited, suggestive in children (0, Ls [29–31, 33, 35, 36, 38, 44, 46]), with 69% of eligible samples showing no association with PA. In adolescents, evidence was limited, non-conclusive (0, Lnc [29, 31, 33, 41, 46]). In adults, findings were inconsistent with limited, non-conclusive evidence (0, Lnc [47]), but experiencing *encouragement from significant others* was perceived as a facilitator to PA in 14 qualitative eligible primary studies in cultural subgroups [26, 39, 42].

Secondly, there was probable evidence that *having a companion for PA* is a determinant of higher PA in children and adolescents (+, Pe [27, 37, 48]) (Table 3, Additional file 4). However, when analysed separately, evidence was limited, non-conclusive in children (0, Lnc [29–31]) and limited, suggestive in adolescents (−, Ls [31, 45]), with 79% of eligible samples showing no association with PA in adolescents. In adults, *having a companion for PA* was inconsistently associated with PA with limited, non-conclusive evidence (0, Lnc [47]) though 73% of eligible samples showed a positive association.

Finally, there was probable evidence that *parental modeling* is not a determinant of PA in children (−, Pe [29, 30]) (Table 3, Additional file 4). In children and adolescents together, it was positively associated with PA in one eligible sample with limited, non-conclusive evidence (++, Lnc [40]). *Parental modeling* was not assessed in adolescents only or adults.

The evidence for the remaining potential determinants from the individuals’ micro-environment at the ‘home/household’, ‘educational institutions’, or ‘neighbourhood’ level was either limited, suggestive or limited, non-conclusive regarding the association with PA across all ages (Table 3, Additional file 4). For example, in children there was limited, suggestive evidence that *parental BMI/waist circumference* was not associated with PA in 80% of eligible samples (−, Ls [29, 30]). In adults, with limited, non-conclusive evidence *neighbourhood satisfaction* was not associated with PA in 88% of eligible samples (−, Lnc [47]).

## Discussion

This umbrella SLR comprehensively summarized and evaluated the current evidence on potential socio-cultural determinants of PA in different age groups. From 23 reviews and over 400 different eligible primary studies, 29 potential determinants were identified. The vast majority (>80%) were assessed in children and/or adolescents and belonged to the micro-environmental home/household level. There was probable evidence that *having a companion for PA* and receiving *encouragement from significant others* were both associated with higher PA in children and adolescents, and that parental *marital status (living with partner)* and experiencing *parental modeling* were not associated with PA in children. In adults, quantitative and conclusive data were scarce. For the majority of potential determinants, evidence on associations was inconsistent and limited.

The fact that a substantial number of reviews was identified highlights socio-cultural determinants of PA as an important area of interest. However, there was a large overlap of eligible primary studies across reviews in youth, revealing that a large proportion of the evidence of potential socio-cultural PA determinants is based on the same data. The overlap in adults was considerably lower, which was mainly due to the focus on subgroups in four out of six reviews [26, 28, 39, 42]. Despite this large overlap especially across the reviews in youth, the conclusion drawn by the reviews with regard to a specific potential determinant was not consistent across the reviews. This was mainly due to heterogeneity in the grading used and in the way of reporting and interpreting results between reviews.

The majority of reviews in our analysis investigated potential determinants of PA in children and/or adolescents, whereas only a minor proportion was on adults. This might be related to the expectation that youths may be more amenable to social influences than adults [49, 50] and, thus, to prevention strategies involving family and peers to foster PA. The fact that PA in childhood may track into adulthood may also be relevant [51]. Knowing (socio-cultural) determinants of PA allows to identify population groups at risk for low PA, which, in turn, might enable counteracting physical inactivity in the youth as an effective strategy to prevent excess inactivity as a risk factor in later life [2]. Further, adult’s PA might be more entrenched and, thus, priority in research on PA modifiability is lower than in youths [51].

In children and/or adolescents, the majority of potential determinants identified belonged to the home/household level, while potential macro-environmental determinants were assessed in adults only. This shift in priority from the micro- to the macro-environmental level from youth to adulthood may be related to the expectation that the influence of significant, individual others (e. g., parents, friends) may decrease as people age, whereas community’s influence may increase (e. g., social expectations, neighbourhood responsibilities) [50, 52].

There was probable evidence that experiencing *encouragement from significant others* was associated with higher PA in children and adolescents. However, this association was only found with probable evidence when children and adolescents were studied together; inconsistent associations with PA were found when focusing on either children or adolescents. Two recent reviews published after our literature search also observed that being encouraged by significant others is positively associated with PA in children and adolescents, which is in line with our findings [12, 53]. Based on models on interactions between parental influences and their offspring’s PA, perceiving being encouraged, supported, or praised increases the offspring’s self-efficacy and perceived competence for PA [11, 54–56]. According to the Social Cognitive Theory, youth’s self-efficacy is recognised as key factor mediating the association between parental support (which was most frequently assessed as *encouragement from significant others*) and their offspring’s PA [57, 58]. Thus, receiving encouragement might have a strong long-term effect. Notably, a close proximity of the source of support might be important [53, 59]. However, the impact of being encouraged might not be strong enough to be constantly observed across all ages. Specifically in adolescence, psychosocial factors inherent to puberty and growing independence might generally attenuate the influence of significant others [50, 52]. Further, the source of influence might shift, with values and behaviours of peers becoming more important than those of family members [50]. Moreover, differences in the definition, assessment, and operationalisation of ‘encouragement’ might have contributed to our inconsistent findings across ages. In this context, a review has shown limited validity and reliability of methods used to assess parental behaviours in studies (including support for PA) [60]. Furthermore, considerable differences in the operationalization and the common use of non-validated methods to assess parental behaviour with regard to their offspring’s PA were shown [61]. In our analyses, the impact of receiving *encouragement from significant others* on PA remained indecisive [11, 62].

*Having a companion for PA* was found to be a determinant of higher PA in children and adolescents with probable evidence. This seems reasonable assuming that a substantial part of PA performed by youths requires teammates, like soccer or playing tag. Unstructured and undirected PA, like free-time play is expected to be relevant for habitual PA in children. Thus, higher PA in children is supposed to be more likely when having a peer for PA; indeed, in a recent SLR published after our literature search, joint participation was positively associated with PA in pre-schoolers [63]. However, when focussing on children or adolescents separately, our analyses revealed inconsistent associations with PA. Again, this might partly be explained by the fact that the effect of *having a companion for PA* is not strong enough to be consistently observed. Further, gender differences might result in inconsistent findings across reviews. Finally, methodological differences in definition, assessment, and operationalisation of this potential determinant might be too heterogeneous to summarize, leading to contradictory results. Interestingly, in adults, 73% of eligible samples included in our umbrella SLR reported a positive association. Thus, one may speculate that among adults, having a companion might again be motivational and promote PA.

Probable evidence was found that parental *marital status (living with partner)* was not a determinant of PA in children, which was also found in adolescents with limited, suggestive evidence. This finding may not be too surprising, given that marital status does not reflect a specific parental characteristic. Thus, parental marital status may encompass several psychological and psychosocial factors that may influence parental behaviour and attitudes, which in turn may or may not have an impact on offspring’s PA. For example, some single parents may have time constraints that limit the time they have available to encourage their children to perform PA, while others compensate for the single-parent status by putting a lot of effort in encouraging. When combining these observations, the overall effect may be null.

Similarly, probable evidence was found that *parental modeling* is not a determinant of PA in children. Again, *parental modeling* per se does not describe a specific parental behaviour but rather describes a generic concept for the way of parenting. Thus, it might not imply whether parental behaviours facilitate or hinder the offspring’s PA. Further, clear definitions of ‘modeling’ were scarce in the reviews included, generally limiting the interpretability. Paucity of evidence prevented an evaluation across the life course, but, *parental modeling* is assumed not to be a PA determinant across all ages.

For the vast majority of potential determinants, no definite conclusion on the association with PA could be drawn. Within the present umbrella SLR, for none of the analysed associations the strength of the evidence was convincing, for some it was probable only. This was due to a lack of consistent results in combination with an insufficient number of reviews and available cohort studies. Further, capturing possible interdependencies between potential determinants and moderators or mediators affecting their association with PA was beyond the scope of this umbrella SLR [59]. Finally, PA as well as potential socio-cultural determinants and their relationship may depend on other factors (e.g., individual, behavioural, etc.). However, the reviews that we identified contained not enough information to further investigate potential effect modification. Therefore, although we only found limited evidence for single potential determinants of PA on a population level, this does not question the existence and importance of socio-cultural determinants in general or rule out the possibility of an impact of potential socio-cultural determinants on PA on the individual level. For example, in adults, in qualitative studies we found taking time out for PA to be perceived as selfish and, thus, as barrier to PA in some cultures [26], while in others it was perceived as being a role model for an active lifestyle and facilitator to PA [42].

There was probable evidence for socio-cultural determinants of PA only at the home/household level for children and adolescents. This finding suggests that the micro-environment is a significant interpersonal influence on PA in younger ages, where one is limited in independent decision making, mobility, and responsibility, providing a crucial domain for promoting youth’s PA. Based on our findings, youths lacking social support should be considered as future target for PA promotion strategies, which is supported by a recent concept mapping DEDIPAC-study that considered supportive factors to have the highest priority in PA research especially in younger ages [64]. Providing opportunities for support and co-activity, thus, might be promising to promote PA. Further, previous studies have shown that parental PA is associated with parental support for PA [56, 65]. Since, in turn, our data suggest that *encouragement from significant others* (encompassing parental support for PA) is associated with PA in youths, fostering parents to be active may be a desirable PA promotion strategy, as it may increase the parental support for PA, which then may promote the offspring’s PA. When focusing only on adolescents, the evidence for potential PA determinants was predominantly limited, suggestive in our umbrella SLR. This could be due to factors related to maturation, which may affect PA as well as potential socio-cultural determinants (e.g., parents reduce supportive behaviour as their offspring matures) or their perception (e.g., being encouraged by parents might be motivational for children, but embarrassing for adolescents). Nevertheless, adolescence warrants attention, since it is a sensitive period for socio-cultural influences [49, 50], with a considerable decrease in PA level also evident during this life stage [50, 52, 66, 67]. Finally, there is need for quantitative data in adults to derive culturally appropriate PA promotion strategies.

As one of seven DEDIPAC umbrella SLRs [15], this umbrella SLR provides an unprecedented comprehensive synthesis of the research on potential socio-cultural determinants of PA in different age groups. A substantial number of reviews and eligible primary studies was included and a broad range of ages and countries of origin was covered. However, our umbrella SLR has some limitations. The majority of eligible primary studies were cross-sectional, making it difficult to infer causal relationships between potential socio-cultural determinants and PA. Cross-sectional studies further bear the risk of reverse causation; for example, the possibility that active youths seek active companions (in contrast to having a companion increases a youths’ PA level) cannot be ruled out. The methodological quality of included SLRs was mostly moderate. However, the AMSTAR checklist is a tool for evaluating the quality of SLRs but not of their primary studies which were the basis for our umbrella SLR [22, 23]. Furthermore, heterogeneity in the measurement, definition, and operationalization, as well as the measurement error in both, PA and potential socio-cultural determinants may have limited our ability to find conclusive evidence on socio-cultural determinants of PA across reviews. Additionally, different PA outcomes and potential determinants were combined, which might attenuate existing associations of determinants with specific PA behaviours. Although the strength and presence of determinants may differ between objectively and subjectively assessed PA [68, 69], it was not possible to take the PA measurement method as criterion into account, when evaluating the evidence on potential socio-cultural determinants, since this information was not systematically provided in the reviews included in our umbrella SLR. However, the aim of this umbrella SLR was to provide a condensed overview of the evidence, and a further division into several types of PA or potential determinants would have had resulted in even less conclusive evidence. For example, when separately examining specific PA outcomes as assessed in the eligible reviews, the findings that *having a companion for PA* is positively associated with PA in children and adolescents and that *parental modeling* is not a determinant of PA in children were no longer present for any PA outcome in our analyses (data not shown). Further, the importance of potential determinants was based on eligible samples as provided by the reviews. Thus, if reviews reported subsamples, one study might be included several times. The requirements for a determinant to have ‘convincing evidence’ for an association with PA were defined very strict and were not achieved. However, concluding ‘convincing evidence’ should be robust against any changes in the near future due to new evidence coming up [25]; especially for socio-cultural determinants, whose relevance is expected to be highly variable depending on the individual and cultural background, this final conclusion should be made cautiously. Due to limited data and the aim to provide a comprehensive overview, sex-stratified analysis were not reasonable. In adults, quantitative data were generally scarce, with no data available regarding ‘workplace’. Finally, the influence of socio-cultural determinants on PA might differ in elderly people; however, no suitable data on older adults (≥65 years [19]) was available.

## Conclusion

In conclusion, out of the 29 potential socio-cultural determinants identified, probable evidence was found that perceiving *encouragement from significant others* and *having a companion for PA* were determinants of higher PA in children and adolescents, while parental *marital status (living with partner)* and *parental modeling* were not determinants of PA in children. No potential determinant showed convincing evidence. The findings should be taken into account when analysing or collecting future epidemiological data. They further help to identify populations at risk for insufficient PA and, thus, to efficiently develop and implement PA promotion strategies. However, although we added new evidence to previous umbrella SLRs on potential PA determinants [9–11] the majority of evidence was inconsistent and inconclusive, not allowing for a definite evaluation of socio-cultural determinants of PA across the life course. Exploring the association of socio-cultural determinants and PA requires further in-depth analyses at the complex interplay of several micro- and macro-environmental influences [59]. For doing so, there is a need particularly for evidence on macro-environmental determinants in children and adolescents, while in adults studies that provide evidence are generally needed.

## Additional files

Additional file 1: Search strategy and key words used for the literature research. Summarizes the search strategy applied in the MEDLINE, Web of Science, Scopus, and SPORTDiscus databases. (XLSX 9 kb)

Additional file 2: Quality assessment of included systematic literature reviews using the AMSTAR checklist assessing eleven quality criteria. Summarizes the results of the methodological quality assessment of included systematic literature reviews based on a slightly modified version of the AMSTAR checklist [22, 23]. Criterion number 11 originally requires the conflict of interest statement in the SLR as well as in the primary studies; for this umbrella SLR the criterion was fulfilled, if the statement was provided in the SLR. *Criteria 1–11 were scored 0, when the criteria was not fulfilled, not applicable, or could not be answered based on the information provided by the systematic literature review, and 1, when the criteria was applicable for and fulfilled by the included systematic literature review. Sum quality score ranged from 0 to 11. **weak (sum quality score ranging from 0 to 3); moderate (sum quality score ranging from 4 to 7); strong (sum quality score ranging from 8 to 11). (XLSX 11 kb)

Additional file 3: Categorization of extracted potential socio-cultural determinants. Summarizes the potential socio-cultural determinants of physical activity (PA) as extracted from the 23 included reviews and as categorized for this umbrella systematic literature review. References indicate the reviews from where the potential socio-cultural determinants were extracted for youths and adults. (XLSX 16 kb)

Additional file 4: Number of reviews, eligible samples, cohorts, and associations for potential socio-cultural determinants of physical activity. Summarizes the number of reviews, eligible samples, and cohorts assessing a specific potential socio-cultural determinant of physical activity (PA) as well as the number of positive, negative, null, or inconclusive associations as extracted from the 23 included reviews. The evaluation of the importance of a potential determinant and the strength of the evidence [24, 25] were based on these results. (XLSX 19 kb)

## Abbreviations

AMSTAR: A MeaSurement Tool to Assess systematic Reviews
ANGELO: ANalysis Grid for Environments Linked to Obesity
Ce: Convincing Evidence
DALY: Disability-Adjusted Life Year
DEDIPAC KH: DEterminants of DIet and Physical ACtivity Knowledge Hub
Lnc: Limited, Non-Conclusive evidence
Ls: Limited, Suggestive evidence
MA: Meta-Analysis
PA: Physical Activity
Pe: Probable Evidence
PRISMA: Preferred Reporting Items for Systematic reviews and Meta-Analyses
SLR: Systematic Literature Review
WHO: World Health Organization
